# A Comprehensive Look at the -13910 C>T LCT Gene Polymorphism as a Molecular Marker for Vitamin D and Calcium Levels in Young Adults in Central and Eastern Europe: A Preliminary Study

**DOI:** 10.3390/ijms241210191

**Published:** 2023-06-15

**Authors:** Magdalena Kowalówka, Grzegorz Kosewski, Daniel Lipiński, Juliusz Przysławski

**Affiliations:** 1 Department of Bromatology, Poznań University of Medical Sciences, Rokietnicka 3 Street, 60-806 Poznań, Poland; grzegorzkosewski@ump.edu.pl (G.K.); jprzysla@ump.edu.pl (J.P.); 2 Department of Biochemistry and Biotechnology, Poznań University of Life Sciences, Dojazd 11 Street, 60-647 Poznań, Poland; daniel.lipinski@up.poznan.pl

**Keywords:** LCT polymorphism, lactase non-persistence, lactase persistence, nutrition, vitamin D, calcium, VDR polymorphism, milk drinking

## Abstract

Intolerance to dairy products resulting from the abnormal digestion of milk sugar (lactose) is a common cause of human gastrointestinal disorders. The aim of this study was to show that the -13910 C>T LCT gene polymorphism, together with genotypes of selected VDR gene polymorphisms and diet and nutritional status parameters, can impact the prevalence of vitamin D and calcium deficiency in young adults. This study was conducted on a group of 63 people, which comprised 21 individuals with primary adult lactase deficiency, and a control group of 42 individuals with no hypolactasia. The LCT and VDR gene genotypes were assessed using PCR restriction fragment length polymorphism (PCR-RFLP) analysis. A validated HPLC method was used to determine serum concentrations of 25(OH)D_2_ and 25(OH)D_3_. Atomic absorption spectrometry was used to determine calcium levels. Their diets (self-reported 7-day estimated food record), estimated calcium intakes based on the ADOS-Ca questionnaire and basic anthropometric parameters were assessed. The CC genotype associated with hypolactasia was found in 33.3% of the subjects. The presence of the CC variant of the LCT gene polymorphism in the study group of young Polish adults was found to be associated with significantly lower milk (134.7 ± 66.7 g/d vs. 342.5 ± 176 g/d; *p* = 0.012) and dairy product consumption (78.50 ± 36.2 g/d vs. 216.3 ± 102 g/d; *p* = 0.008) compared with lactase persistence. At the same time, people with adult-type primary intolerance were found to have statistically significant lower serum levels of vitamin D and calcium (*p* < 0.05). There was a higher chance of vitamin D and calcium deficiency and a lower intake in the group exhibiting lactase non-persistence (OR > 1). The AA variant of the VDR gene’s BsmI polymorphism present in people with hypolactasia may further contribute to an increased risk of vitamin D deficiency. Exclusion of lactose from the diet, combined with impaired vitamin D metabolism, may also lead to inhibited calcium absorption by the body. Further research should be carried out on a larger group of subjects to clarify the relationship between lactase activity and vitamin D and calcium levels in young adults.

## 1. Introduction

Lactose digestion disorders, as well as milk and dairy product intolerance, are among the most common human gastrointestinal dysfunctions. Lactase non-persistence is a worldwide phenomenon, but it affects people with varying degrees of severity. According to estimates, between 31 and 37% of adults in Poland are affected [1,2,3].

Lactose is the main and unique carbohydrate in milk. It is also present in all milk products. Lactose may also be an additive in a variety of foods, such as pastries and confectionery, ice creams, breads, frozen meals, cured meats, powdered soups or margarine. Some spices, sweetening agents, aromas and chewing gums contain small amounts of lactose. Furthermore, milk sugar is one of the most popular and versatile filling agents and excipients used for tableting and encapsulation within the pharmaceutical industry [4,5,6]. It is used as a carrier for the actual active substance in the drug manufacturing process. 

This milk sugar is the main source of energy for newborn mammals. It also reduces appetite. Lactose is involved in the absorption and retention of important minerals such as calcium, magnesium and zinc. It is also the only dietary source of galactose, essential for synthesizing macromolecules such as oligosaccharides, glycoproteins and glycolipids, which are used as components of nerve cell membranes [7]. Lactose exhibits probiotic properties and thus has a beneficial effect on the functioning of the intestinal epithelium and the intestines themselves [8,9]. The endogenous enzyme lactase-phlorizin hydrolase known as lactase (LCT) is required to digest lactose into monosaccharides. There are three main types of lactase deficiency:

- Alactasia, caused by a lack of lactase in the body (rare);
- Secondary lactase deficiency, either transient or chronic, depending on the type and duration of the agent damaging the small intestinal mucosa;
- Primary lactase deficiency, which manifests itself in adolescence or early adulthood [10,11,12].

In primary lactase1 deficiency, the activity of the enzyme responsible for breaking down milk sugar decreases with age. This is a natural condition called adult type hypolactasia (ATH) or lactase non-persistence (LPN). It is a genetically determined type of reduced lactase [13,14,15,16]. There are two distinct phenotypes: a persistently high enzymatic activity of lactase throughout a lifetime and one where its expression decreases with age due to a decrease in precursor protein synthesis in epithelial cells.

Lactase deficiency in adults may be caused by a recessively inherited polymorphism of the LCT gene. The lactase persistence phenotype is inherited as an autosomal dominant trait [17,18,19].

Within the European population, the persistence or non-persistence of lactase expression is primarily closely related to a single nucleotide polymorphism (SNP), LCT-13910 C>T (rs4988235), which is located in the promoter region of the gene encoding LCT. This polymorphic variant occurs as a CC, CT or TT genotype [13,20,21]. The CC genotype is a good predictor of reduced intestinal lactase expression, whereas the TT genotype is a predictor of persistently high enzymatic expression. 

The CT genotype is characterized by intermediate levels of lactase expression, which are usually sufficient to digest lactose [22,23,24].

**Figure 1:**
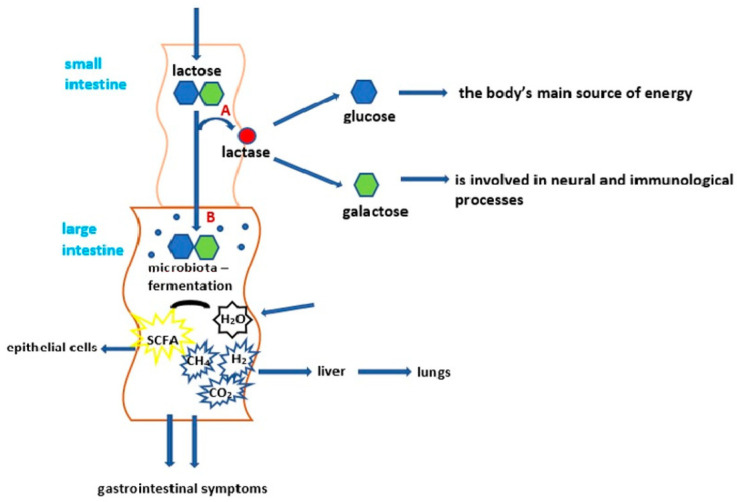
Diagram of lactose digestion during lactase persistence (A) and lactase non-persistence (B) modified from [12].

Reduced or absent lactase expression, and the consequent presence of milk sugar in the intestines, causes an increase in osmotic pressure in the gut, leading to the occurrence of diarrhea (stools exhibit a characteristic sour smell), abdominal pain and sometimes nausea and vomiting [25,26] (Figure 1).

Genetic factors, as well as ethnicity, impact the prevalence of primary lactose intolerance. Looking at Europe, ATH affects 5% of white adults in Great Britain, 6% in Denmark, 15% in Germany, 17% in Finland and northern France and up to 40% in Mediterranean countries [27]. On the other hand, the figures for South America, Africa and Asia are much higher, with more than 50% of the population suffering from this type of condition; in some Asian countries it is even close to 100% [28,29]. 

Dietary history and finding an association between the diet and the dyspeptic symptoms play an important role in diagnosing ATH. 

Excluding milk and its products from the diet can lead to calcium and vitamin D deficiency. Calcium homeostasis is affected by vitamin D status, calcitriol production and nuclear vitamin D receptor (VDR) expression. Vitamin D increases the absorption of calcium and phosphate from the gastrointestinal tract. It supports bone ossification and mineralization and optimizes the mineral density of bones. It is essential for the proper functioning of the nervous, muscular, immune and endocrine systems [30,31]. 

The VDR receptor, a member of the nuclear steroid hormone receptor family, is responsible for the pleiotropic effects of vitamin D. The function of the VDR gene is determined by its polymorphism. Polymorphic variants in genes encoding proteins involved in vitamin D metabolism and transport can affect the levels of that vitamin in the body [32,33]. There are four main VDR gene polymorphisms. Some of its SNPs may contribute to reduced vitamin D concentrations; analysis of the serum levels of this vitamin and its metabolites may be relevant for many diseases [34].

In humans, vitamin D occurs in two main inactive forms: vitamin D_2_ and vitamin D_3_, of which vitamin D_3_ accounts for more than 90% of the total vitamin D level [35]. Cutaneous synthesis and diet are the primary sources of vitamin D for humans. As a result of hydroxylation in the liver, inactive vitamin D is converted to the metabolite 25(OH)D, which is a vitamin D status biomarker [36]. According to guidelines, 25(OH)D < 20 ng/mL is defined as vitamin D deficiency and 20–30 ng/mL 25(OH)D is suboptimal, while the optimal vitamin D concentration is 25(OH)D ≥ 30 ng/mL [37,38].

This preliminary study was carried out to verify the hypothesis that the -13910 C>T LCT gene polymorphism in association with selected variants of the VDR gene, which encodes the vitamin D receptor, as well as diet and nutritional status, may influence the occurrence of vitamin D and calcium deficiency in the body and the pathogenesis of diseases associated with these deficiencies in young adults.

## 2. Results

**Figure 2:**
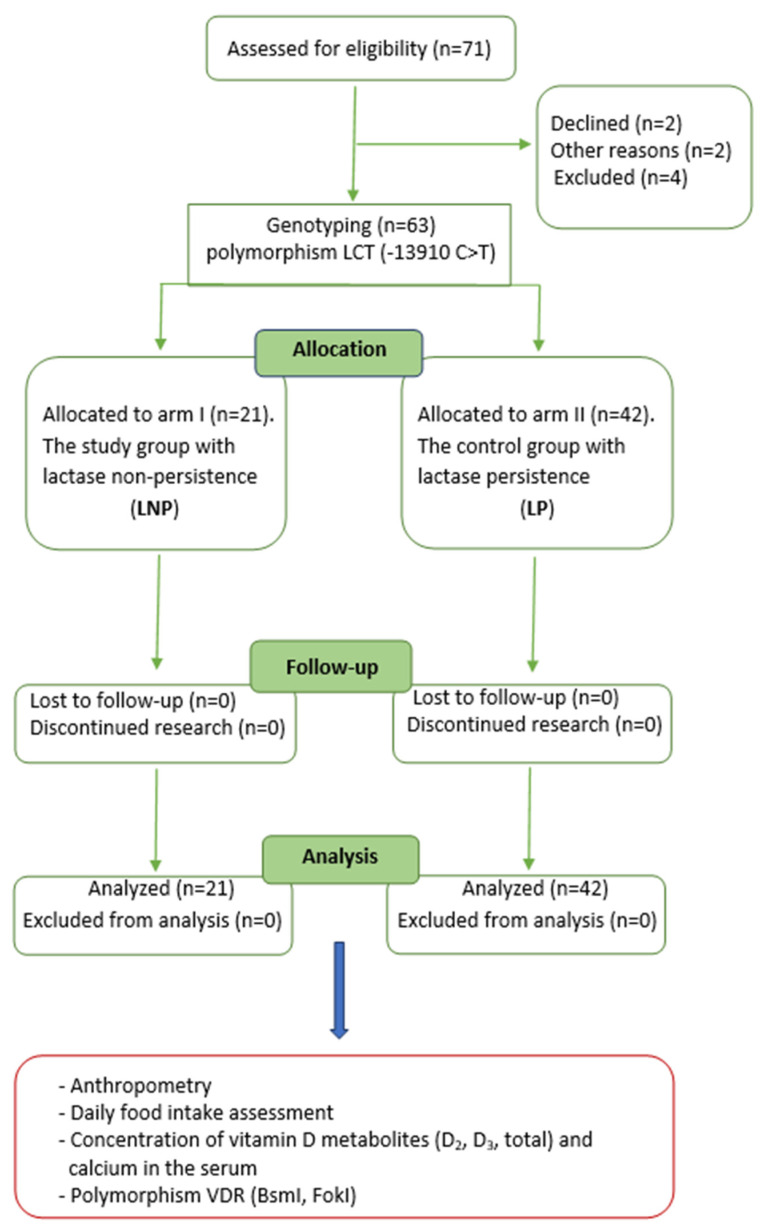
Flowchart of the study.

A flow chart of this study is shown in Figure 2. Out of the seventy-one people included in the study, eight did not meet the designated criteria. All the remaining subjects (n = 63) were genotyped for the LCT polymorphism (-13910 C>T).

**Figure 3:**
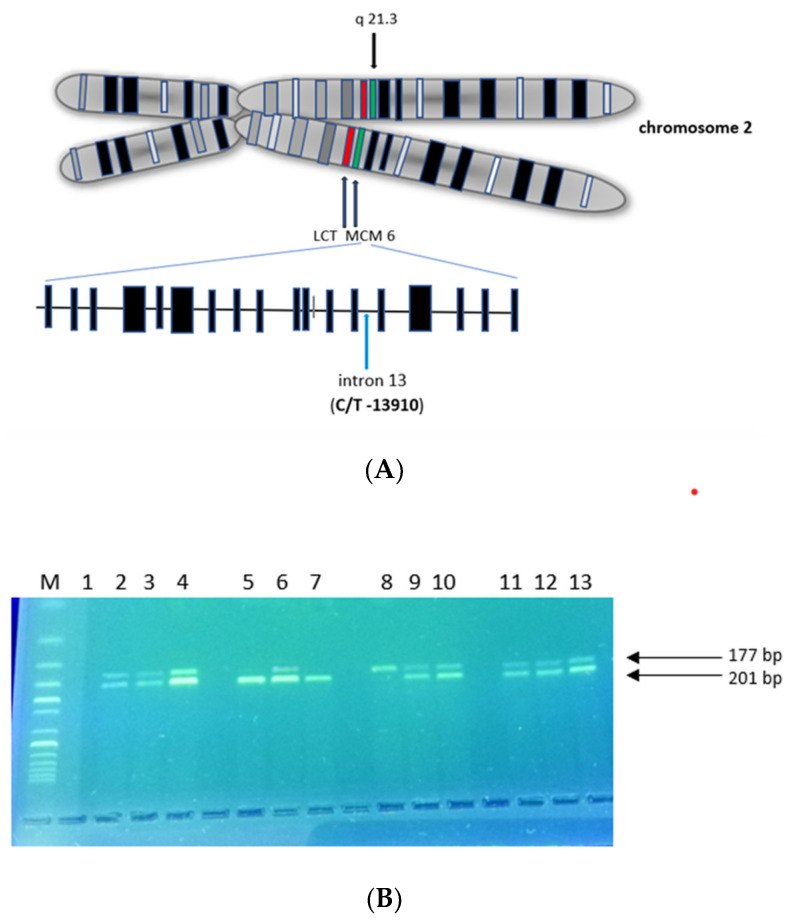
(**A**) Polymorphism in LCT gene (-13910 C>T), red of box—locus LCT gene, green—locus MCM 6 gene; (**B**) PCR-RFLP-based genotyping. Path M: DNA marker GPB1000 bp; path 1: negative control; paths 5,7: homozygote CC; paths 2,3,4,6,9,10,11,12,13: heterozygote CT; path 8: homozygote TT.

The gene encoding lactase is located on the long arm of chromosome 2 in position 21.3 (2q21.3). The ability to produce lactase is determined by the presence of the -13910 C>T polymorphism. The variant is located in a non-coding region in the genome in intron 13 of the minichromosome maintenance type 6 gene (MCM6) [39] (Figure 3A). A change of the single nucleotide C to T in position -13910 significantly affects LCT transcription.

Amplification of the promoter fragment of the LCT gene encoding lactase resulted in a 201 bp product for the polymorphism in question. The result of hydrolysis of the PCR product was revealed in an agarose gel with the *HinfI* restriction enzyme. Larger DNA fragments corresponded to C alleles, while smaller ones corresponded to T alleles (or, more precisely, to the larger product of hydrolysis (177 bp), as the smaller one (24 bp) was not visible in agarose gel. Genotypes were identified by the presence or absence of an appropriate restriction site. Recessive homozygote (CC) was characterized by the presence of a single strand (201 bp) (Figure 3B).

Table 1 shows the obtained allele and genotype frequencies for the study group. Those with the CC genotype, indicating hypolactasia, accounted for 33.3% (n = 21); 50.8% had CT heterozygotes (n = 32); 15.9% (n = 10) had TT homozygotes. The CT and TT variants were associated with a lactase persistence phenotype (normolactasia).

For the CT genotype, lactase deficiency is rarely identified, as this is due to compensation for the presence of the lactase persistence (T) allele, which has a dominant effect. Hence, it is believed that most heterozygous individuals produce sufficient lactase.

The C and T allele frequencies in this study group were 58.7% and 41.3%, respectively. 

There was no statistically significant difference between the expected genotype and allele values and the actual values for the subjects (*p* > 0.05); genotype frequencies were within the Hardy–Weinberg equilibrium (χ^2^ < 3.46, α = 0.05). The frequencies of the investigated alleles and genotypes of the LCT gene were consistent with data published in the NCBI SNP database [40].

Those with the CC genotype were allocated to the LNP study group (arm I) and those with the CT and TT variants (n = 42) were assigned to the LP control group (arm II) (Figure 2). 

The mean values and medians for the selected anthropometric parameters are shown in Table 2. Women comprised 63% of the study group and men made up the remaining 37%. No relationship between genotypes and gender was found. The groups were homogeneous in terms of age. Study participants were between 21 and 31 years old and the mean median age of young adults in the LNP group was 23.0 ± 1.50 years and 24.0 ± 1.00 years for those in the control group. Body weights were between 47.4 kg and 93.3 kg. There were no significant differences in age, height or body weight between the groups.

Indicators of nutritional status were assessed based on the performed anthropometric analysis. Mean BMI was approximately 23 kg/m^2^ for both study groups (BMI in the LNP group was between 17.6 kg/m^2^ and 28.4 kg/m^2^; in the LP group it was between 18.8 kg/m^2^ and 31.0 kg/m^2^). According to the norms for this index, the majority of the subjects had a normal body weight. 

Despite lower bone mass in those with impaired ATH, there was no statistically significant difference compared with the control group (*p* > 0.05). In addition, there were no significant differences for body fat (%) and water (%) (Table 2). 

Estimated total energy and protein intakes were similar: 2033 ± 542 kcal/d for lactase non-persistent subjects and 2094 ± 499 kcal/d for healthy subjects (Table 3). Daily protein intakes were also similar at 85.8 ± 27.4 g/d vs. 84.1 ± 21.9 g/d; 17% of energy was obtained from protein. A lower intake of fats was shown for the LNP group (68.8 ± 16.8 g/d) compared with the control group (80.3 ± 29.5 g/d); however, no significant differences were observed (*p* > 0.05). A total of 32.7 ± 4.42 % kcal of energy was obtained from fat in the ATH group and 34.3 ± 5.32 % kcal from fat in the LP group. Carbohydrate intakes were at similar statistically non-significant levels in both participant groups subject to analysis (253 ± 60.3 vs. 262 ± 66.8 g/d). 

The total vitamin D intake for those with hypolactasia was 2.32 ± 0.36 µg and was significantly lower compared with those with CT and TT variants (4.57 ± 0.50 µg) (*p* = 0.045). 

Calcium intake was also found to be lower in the group with the CC genotype (785 ± 97.5 mg/d). In healthy subjects, the calcium intake was at 881 ± 128 mg/d (Table 3); this difference was statistically significant (*p* = 0.048). 

Unfortunately, the intake of calcium and vitamin D for the majority of the subjects studied was below the estimated average requirement (EAR) in Poland. 

No statistically significant differences were found with regard to phosphorus intake in the groups subject to analysis.

An assessment of the intake of milk and dairy products, as well as calcium from dairy products, was also carried out using the ADOS-Ca questionnaire. 

There was a significantly (*p* = 0.008) lower intake of milk (78.50 ± 36.2 g/d) and dairy products (134.7 ± 66.7 g/d, *p* = 0.012) for people with reduced lactase activity (LNP) compared with the control group (342.5 ± 176; 216.3 ± 102 g/d, respectively) (Table 4).

**Figure 4:**
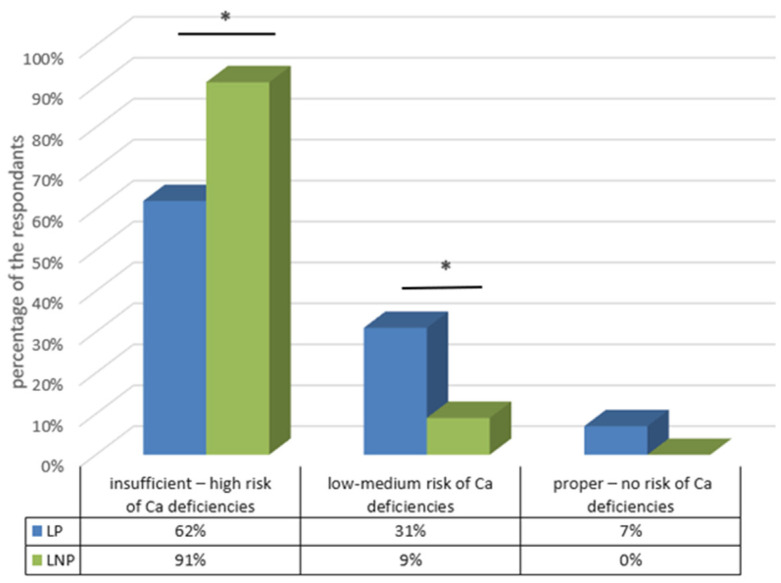
Estimated calcium intakes based on the ADOS-Ca questionnaire in the study group. Statistically significant differences (*p* < 0.05) are marked with an *.

In the study population, the prevalence of inadequate calcium intake and the associated high risk of calcium deficiency among those with identified hypolactasia was statistically significantly (*p* < 0.05) higher (91%) than for those in the LP group (62%). In addition, 31% of the control group had a low calcium intake and 7% of the subjects showed a normal intake. A total of 9% of those with the CC genotype had an average risk of calcium deficiency and no one in the LNP group showed a normal intake (Figure 4). 

Analysis of serum biochemical parameters in an unadjusted model showed significant differences in 25(OH)D_3_ metabolite (17.6 ± 2.19 ng/mL, *p* = 0.025) and total 25(OH)D levels (18.1 ± 2.91 ng/mL, *p* = 0.022) in hypolactasia vs. TT variant subjects (21.5 ± 3.99 ng/mL and 22.6 ± 4.46 ng/mL, respectively).

Furthermore, total calcium levels were also significantly lower (2.27 ± 0.29 mg/dL, *p* = 0.049) for those with the CC genotype compared with those with the TT genotype (2.62 ± 0.26 mg/dL) (Table 5).

**Figure 5:**
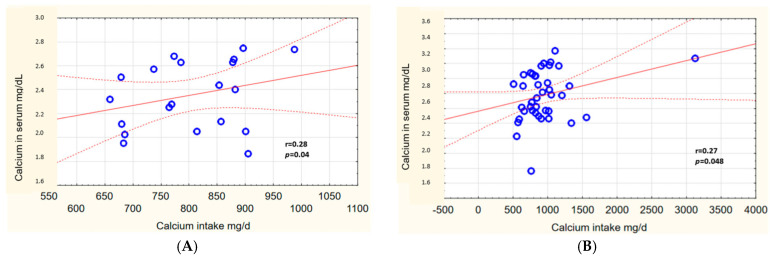
Scatter plot of variables: relationship between calcium intake and its concentration in blood serum in a group of people with hypolactasia (**A**) and control group (**B**). The red line is a regression line with a 95% confidence interval, bounded by dashed lines. The blue circles are the values of the analyzed variables.

**Figure 6:**
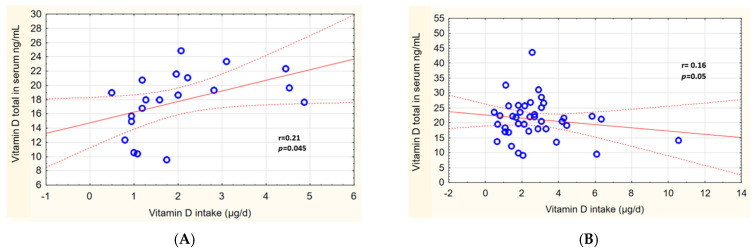
Scatter plot of the studied variables: dietary vitamin D intake and serum vitamin D concentration (total) in subjects with hypolactasia (**A**) and in the control group (**B**). The red line is a regression line with a 95% confidence interval, bounded by dashed lines. The blue circles are the values of the analyzed variables.

Analysis of the mutual relations between calcium intake from diet and serum concentrations showed a weak positive correlation in the hypolactasia group (r = 0.28) and in the control group (r = 0.27) (Figure 5A,B). A low positive correlation between vitamin D intake and serum vitamin D concentration (total 25(OH)D) was identified for those with ATH (r = 0.21) (Figure 6A).

A multivariate logistic regression model showed a significant relationship between impaired lactose tolerance due to the CC variant of the lactase gene polymorphism and serum levels of calcium and vitamin D (total) metabolites and their intake (Table 6). A higher chance of vitamin D and calcium deficiency and lower intake in the group exhibiting lactase non-persistence compared with the control group was demonstrated (OR > 1). 

Table 7 shows the allele and genotype frequencies of the VDR gene polymorphism SNPs, BsmI (1024 + 283 G > A, rs1544410) and VDR FokI (c.2T > C, rs2228570), in the study group. The BsmI polymorphism was shown to have a G allele frequency of 50.8% and an A allele frequency of 49.2%. In contrast, for the FokI polymorphism, the frequencies of T and C alleles were 38.9.2% and 61.1%, respectively. The frequencies of the investigated alleles and genotypes of the VDR gene were consistent with data published in the NCBI SNP database [40].

**Figure 7:**
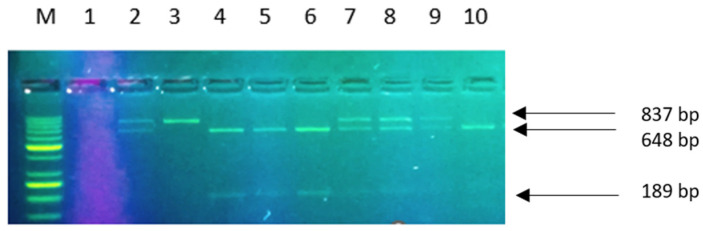
PCR-RFLP-based genotyping in VDR genes (BsmI). Path M: DNA marker GPB1000 bp; path 3: homozygote GG; paths 2,7,8,9: heterozygote GA; paths 4,5,6,10: homozygote AA; path 1: negative control (-), where with proper cleanliness work, no product will be created, (most often without matrix).

**Figure 8:**
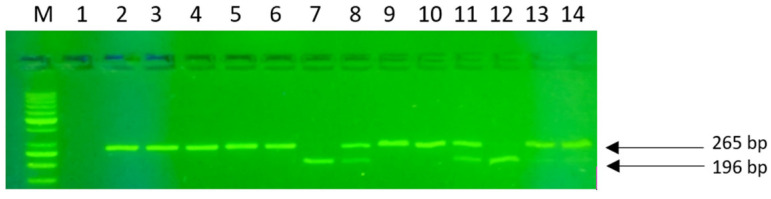
PCR-RFLP-based genotyping in VDR genes (FokI). Path M: DNA marker GPB1000 bp; paths 2–6,9,10: homozygote TT; paths 8,11,13,14: heterozygote TC; paths 7,12: homozygote CC; path 1: negative control (-).

Figure 7 and Figure 8 show the obtained genotyping products after restriction enzyme digestion (RFLP) and agarose gel electrophoresis. 

Rs1544410 (BsmI) genotyping showed no statistically significant differences in the distribution of VDR gene polymorphism genotypes between the LNP group and the healthy subjects (Table 8). In the study population, the GA genotype of the BsmI polymorphism was dominant in both the LNP and LP groups (47.6% vs. 57.2%). 

For the FokI polymorphism (rs2228570), it was shown that individuals with hypolactasia were mainly characterized by the TC (42.9%) and TT (38.1%) genotypes. The CC variant was dominant in LP subjects (42.9%), while the impaired lactose tolerance genotype was least common (19.0%). This difference was statistically significant (*p* = 0.042) (Table 8).

In the study groups, the frequencies of BsmI and FokI polymorphism genotypes were within the Hardy–Weinberg equilibrium (χ^2^ < 3.46, α = 0.05) (Table 9). 

Analysis of vitamin D (total 25(OH)D) levels and serum calcium levels in subjects with hypolactasia showed that carriers of the AA genotype of the VDR gene BsmI polymorphism had statistically significantly lower levels (10.4 ± 1.25 ng/mL, *p* = 0.017 and 2.28 ± 0.12 mg/dL, *p* = 0.049, respectively) compared with those with the GG genotype. A similar relation was found for lactose-tolerant individuals. Individuals with the GG genotype had significantly higher (*p* = 0.048) serum vitamin D levels (22.6 ± 3.53 ng/mL) than those with the AA variant (17.0 ± 2.79 ng/mL) (Table 10). 

Analysis of the FokI polymorphism among individuals with LNP showed the lowest serum vitamin D (13.2 ± 2.16 ng/mL) and calcium (2.04 ± 0.06 mg/dL) concentrations among those with the TT genotype compared with the TC and CC variants. The obtained differences were at the threshold of statistical significance (*p* = 0.05). For lactose-tolerant individuals, there were no significant differences between vitamin D and calcium levels for different FokI genotypes (Table 10).

**Figure 9:**
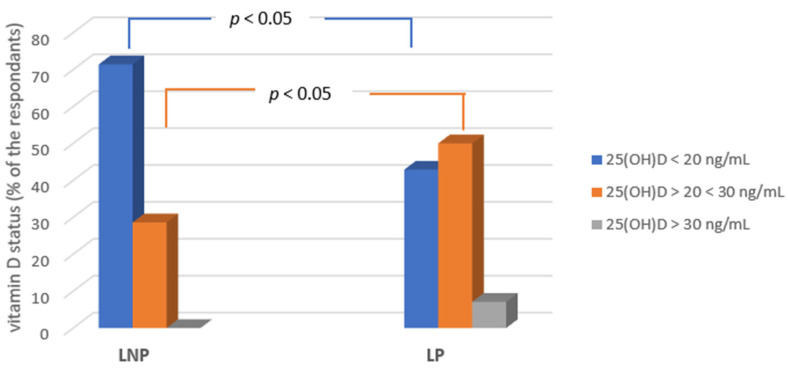
The percentage of people with vitamin D_2_ + D_3_ deficiency in patients depending on lactase persistence and non-persistence.

Lactase-deficient participants had significantly lower (*p* < 0.05) total 25(OH)D concentrations compared with the milk sugar-tolerant group. A total of 71% of subjects had severe vitamin D deficiency and 29% had suboptimal concentrations 20 < (25(OH)D < 30 ng/mL. For the control group, 40% of the subjects had a vitamin D deficit and 10% had optimal concentrations (Figure 9). 

There were no significant differences within the scope of body weight, BMI and bone mass and basal dietary components between the study groups (LNP vs. LP) in relation to vitamin D status. Serum vitamin D levels appeared to be partially dependent on vitamin D intake. The group with hypolactasia and severe serum vitamin D deficiency (<20 ng/mL) had the lowest intake of this vitamin (2.23 ± 0.72 µg/d). The obtained differences were at the threshold of statistical significance (*p* = 0.05). Participants with severe vitamin D deficiency were also observed in the lactose-tolerant group; however, their vitamin D intake was higher (3.92 ± 1.22 µg/d). In addition, individuals with optimal serum vitamin D levels and the highest intake (>30 ng/mL, 5.13 ± 0.89 µg/d) were identified. The resulting differences in vitamin D intake according to vitamin D supply status in the LP group were statistically significant (*p* = 0.048) (Table 11).

Individuals with hypolactasia and severe serum vitamin D deficiency predominantly had the GA (42.9%) and the AA genotypes (23.8%) of the VDR gene BsmI polymorphism. For the FokI polymorphism, it was shown that more people with ATH and deficient vitamin D levels had the TT genotype (33.3%). LP individuals and those with higher serum vitamin D concentrations were mainly of the CC and TC genotype (Table 11). 

## 3. Discussion

Research on lactase phenotypes has been ongoing for many years. Despite many studies, the molecular mechanisms that determine the development of lactase deficiency with age are still not understood completely.

Today, modern molecular biology diagnostic methods make it possible to identify the genetic basis of this condition.

This study analyzed polymorphic variants of the LCT gene associated with different lactase activity and their possible relationship with serum vitamin D and calcium levels, diet and nutritional status, as well as the vitamin D receptor promoter genotype (BsmI and FokI), in a group of young Polish adults.

A total of 33% of the study population had the CC genotype and reported gastrointestinal symptoms of varying severity after consuming dairy products. The frequencies of genotypes obtained in the study were within the Hardy–Weinberg equilibrium and were consistent with previous studies performed by other facilities [1]. 

Overall, it is estimated that around two-thirds of people worldwide have a problem digesting lactose in adulthood [18]. 

Suppressive transcriptional epigenetic changes (e.g., DNA methylation) that accumulate over time and lead to decreased lactase levels in adulthood impact the severity of the symptoms associated with hypolactasia [41]. The presence of the T allele means that age-dependent DNA methylation is not significant and does not reduce lactase levels in adulthood [42,43]. 

Hypolactasia depends not only on lactase expression but also on the amount of lactose in the diet, intestinal flora, gastrointestinal motility, small intestinal bacterial overgrowth and the sensitivity of the gastrointestinal tract to the production of gas and other lactose fermentation products [44]. Individuals with adult-type hypolactasia have a significantly lower clearance of short-chain fatty acids, i.e., propionate, acetate and butyrate (SCFA). In patients with ATH, lactose intake of more than 12 g is estimated to contribute to adverse symptoms due to milk sugar consumption, whereas low lactose intake is most often not a problem [45,46,47,48].

The ability to ferment undigested lactose depends mainly on the composition of the colonic and intestinal microbiota. A positive correlation between the occurrence of hypolactasia and probiotics of a specific strain and concentration has been demonstrated [49]. Alleviation or exacerbation of lactase deficiency symptoms has been observed depending on the abundance of certain bacterial strains in the colon. Colonic bacteria effective in lactose fermentation help to reduce osmotic shock which causes diarrhea but can result in an increased production of gasses. Interestingly, heterozygous carriers of LCT-13910 C>T and LCT-22018 G>A showed intermediate enzymatic activity causing symptoms of lactose intolerance in stressful situations or during intestinal infections [12]. 

The research findings we presented applied to:

### 3.1. Potential Differences in Anthropometric Characteristics

Nutritional status parameters for individuals with lactase non-persistence were not significantly different from the control group. Bone mass was lower in those with the CC genotype, but the difference was not significant. In contrast, Mnich et al. [50], in their study, demonstrated a significant correlation between lower bone density and the CC genotype. It was also noted that individuals with lactase deficiency were more likely to suffer from osteoporosis, however these values were not statistically significant compared with individuals with CT and TT genotypes [50]. Popadowska et al. [51] did not show an association between the -13910 C>T LCT gene polymorphism and obesity. However, a subsequent study by Popadowska and Kempinska-Podhorodecka [52] revealed that the CC genotype was linked with a reduced intake of milk and dairy products, as well as higher lean mass and larger forearm circumference, which may have implications for dietary management of ATH. 

According to Alharbi and El-Sohema [43], individuals with the CC genotype were shorter than those with the TT genotype; presumably, as confirmed by numerous studies, this determined lower milk consumption [53,54,55,56]. 

### 3.2. Vitamin D and Calcium Intake vs. Their Serum Concentrations

Multivariate analysis showed that adult-type hypolactasia was associated with a higher risk of vitamin D and calcium deficiency, as well as lower calcium intake, compared with lactase-persistent (LP) individuals.

Vitamin D intake in the Polish population has been very low for years and is far from meeting dietary recommendations, which have been set at an adequate intake (AI) level of 15 µg/day [57]. For adults, the intake is within the range of 1.4–5.1 µg per day. Hence, according to recent recommendations, depending on body weight and dietary vitamin D supply, year-round vitamin D supplementation may be recommended in Poland [58]. 

The mean intake of vitamin D in other European countries is also low (less than 5 μg/day (200 IU/day) in most countries). It is highest in Scandinavian countries and also for the Innuit population due to the consumption of oily fish and cod liver oil and fortified dairy products [59]. 

The relationship between the LCT-13910 C>T polymorphism genotypes and the consumption of milk and dairy products reflected with high probability the vitamin D and calcium serum levels in the subjects. The mean concentrations of the vitamin in question in the LNP and LP groups were found to be below the recommended figures, but those in the LNP group had statistically significantly lower hypovitaminosis D (*p* < 0.05) compared with those with the TT variant. A similarly significant relationship was shown for calcium concentrations. The mean calcium level for individuals with diagnosed hypolactasia was at the lower limit of the recommended range, as opposed to those with the TT genotype. Vitamin D plays an essential role in maintaining phosphorus and calcium homeostasis and in stimulating bone mineralization. Calcium deficiency in lactose-intolerant individuals is often associated with lower bone mineral density [60,61]. In a Canadian population study, Alharbi and El-Sohemy [43] also demonstrated lower serum vitamin D levels due to reduced dairy product intake. 

Milk and dairy products are the richest source of easily assimilated calcium in the diet, mainly due to the presence of lactose, milk phosphopeptides and a favorable 1.4:1 calcium: phosphorus ratio. They are the main source of calcium in the diets of Poles and other Europeans, providing between 60% and 80% of the total amount [57]. Calcium homeostasis in the human body depends on vitamin D status, the production of 1,25(OH)2D_3_ and the expression of the nuclear receptor VDR, which is known to regulate the expression of genes important for calcium balance and bone metabolism, as well as through the presence of other absorption promoters. Calcium in dairy products reduces fat absorption and may therefore prevent the development of cardiovascular diseases [62]. At the same time, it has been suggested that calcium deficiency may be a factor which increases the risk of obesity through excessive fat accumulation and contributes to the development of type 2 diabetes [62]. The benefits associated with dairy product consumption also include improved hypertension control, weight gain and reduced risk of colorectal cancer.

The research carried out within the scope of this study showed a statistically significantly lower calcium intake in the LNP group subjects (*p* < 0.05). The demonstrated lower total intake of milk and of dairy products for people with reduced lactase activity compared with the control group may be the cause of this unfavorable phenomenon. 

The application of the ADOS-Ca questionnaire enabled a more accurate characterization of this intake in the study population by distinguishing those with inadequate, low and correct recommended intake levels of this mineral. Through this, it was found that study participants who avoided eating milk and other dairy products in particular due to symptoms associated with the presence of hypolactasia were at a greater risk of calcium deficiency. 

This was in line with a number of other studies. Di Stefano et al. demonstrated that lactose-intolerant subjects consumed statistically significantly less calcium in their diet compared with lactose-tolerant subjects [63]. Furthermore, young adults with lactase deficiency exhibited elevated parathormone (PHT) levels [64]. Koek et al. [65] demonstrated a clear relationship between dietary calcium intake and serum ionized calcium levels. However, Yahya et al. [66] showed that, although young Malaysian adults had a high prevalence of ATH, there was no direct effect on bone health, unlike calcium intake, which was low. Enattah et al. [24] showed that hypolactasia and abnormal lactose digestion did not alter calcium absorption and bone turnover rates nor did it interfere with reaching peak bone mass. 

### 3.3. Assessment of LCT and VDR Gene Polymorphisms Interactions

Our results allowed us to assess gene interactions between lactase gene polymorphisms and genetic VDR gene changes, as well as the effect of polymorphism on vitamin D status. The 1.25(OH)2D_3_ encoding VDR gene activates a rapid receptor binding to regulatory regions of target genes and causes changes in transcription [67]. Expression of vitamin D receptors is associated with the occurrence of polymorphisms in the VDR encoding gene. The most common polymorphisms include the morphs referred to as FokI, BsmI, TaqI and ApaI. These polymorphisms may affect vitamin 25(OH)D serum levels [68]. The vitamin D receptor gene mediates the action of the hormone system in calcium homeostasis and the VDR genotype also impacts the gut’s ability to absorb calcium. In relation to the pleiotropic effects of vitamin D, correlations have been demonstrated to exist between VDR polymorphisms and various diseases such as insulin resistance, type 2 diabetes, abdominal obesity and responses to calcium and vitamin D supplementation [69]. Vitamin D receptor polymorphisms may be the reason for why not all individuals benefit from vitamin D supplementation [70]. 

Of the many polymorphisms of the VDR gene described, two affect VDR molecular signaling. These are BsmI, located in the gene intron sequence and FokI in the encoding section. Changes in VDR expression associated with polymorphisms impact vitamin D-dependent functions. Individuals with the GG genotype (BsmI) exhibit increased plasma concentrations of 1α,25-dihydroxyvitamin D_3_ [71].

Analysis of serum vitamin D and calcium levels according to the study group (LNP and LP) and VDR genotype showed that subjects with adult type hypolactasia had significantly lower levels when it came to carriers of the AA BsmI genotype compared with carriers of the GG variant in both study groups, who exhibited the highest 25(OH)D and calcium levels. A similar relation was shown by Abouzid et al. [34], where levels of 25(OH)D_3_ and 3-epi-25(OH)D_3_ were significantly higher for the GG genotype as compared to the GA variant.

For individuals with lactase deficiency (LNP) and the TT variant of the FokI polymorphism, we demonstrated the lowest statistically significant vitamin D and calcium levels. A similar relation was not found for lactose tolerant individuals. 

Divanoglou [72] suggested that epigenetic VDR modifications regulate the conversion of vitamin D to its metabolites via CYP450, thereby affecting vitamin D concentrations. Santos et al. [73] demonstrated the association of wild-type BsmI, ApaI and TaqI variants of the VDR gene with low 25(OH)D levels. However, these results were not consistent. In contrast, Cobayashi et al. [74] observed that it was the presence of the mutant A allele of the BsmI polymorphism that constituted an increased risk of vitamin D deficiency. Valtuena et al. [75] showed no association between the BsmI polymorphism and vitamin D concentrations in adolescents under 18 years of age. Furthermore, according to Jakubowska-Pietkiewicz et al. [76], the BsmI and FokI polymorphisms of the VDR gene did not directly impact the calcium–phosphate metabolism in young individuals. Discrepancies between different study populations can be partly explained by ethnic, geographical and genetic differences.

In order to alleviate the symptoms resulting from impaired lactase activity, sufferers often choose to eliminate milk and dairy products from their diets. This action exposes them to a risk of both vitamin D and calcium deficiencies; however, only congenital lactase deficiency requires complete elimination of milk sugar from the diet. Hypolactasia does not require complete elimination of lactose from the diet, only a reduction of its intake to approximately 10–12 g/day [12,77]. Some probiotics can improve the digestion of lactose and thus alleviate dyspepsia symptoms. Therefore, fermented dairy products can still be consumed by people with ATH as they exhibit high β-galactosidase activity, partial lactose hydrolysis and slower intestinal transit times. This is also true for maturing hard cheeses and brie-type cheeses. It is only recommended to limit the consumption of milk in its pure form to 50–100 mL, depending on individual sensitivity to milk sugar [6].

Lactose tolerance may be increased by taking probiotics, which alter the colonic microflora. Furthermore, in people with LNP, a continuous intake of small amounts of lactose leads to the microbiome adapting, resulting in altered metabolomes [78,79]. 

Today, more and more reduced-lactose products are becoming available. An enzymatic lactose hydrolysis process is usually applied to such products. The market for this type of dairy product is the fastest growing dairy industry sector. However, the lactose hydrolysis process increases the cost of producing milk and dairy products, which translates into a higher price on the shelves.

#### Limitations

The main limitation of the present study was the relatively small sample size, which probably resulted in other relationships between genotyping and environmental factors not being revealed. However, this was a preliminary study. Financial issues and the limited time available to conduct the survey also made it difficult to put together a large study group.

Furthermore, the genotyping results obtained did not exclude the possibility that the risk of vitamin D and calcium deficiency could also be affected by other SNPs in the VDR gene. Therefore, further assessments of vitamin D status and calcium levels are required. This was also true for genotyping other major VDR polymorphisms, such as TaqI, ApaI and Cdx-2. The study population was not divided into male and female groups, since genotyping was not shown to be related to gender and the sample size was small (although the calculated minimum sample size was reached). This perhaps meant that significant differences in levels and intakes of vitamin D and calcium, as well as the other parameters studied, were not demonstrated. In addition, the samples for the present study were taken in autumn and winter, so the seasonal variation in vitamin D levels in the body, associated with cutaneous synthesis, was not taken into account.

The strengths of the study included the fairly high homogeneity of baseline results between the analyzed groups of people with lactase non-persistence and lactase persistence, as well as the use of an ethnically homogeneous population and the use of multiple predictors to determine the intake, as well as serum vitamin D and calcium status. 

## 4. Materials and Methods

### 4.1. Design of the Study

The study was carried out on a group of 71 subjects recruited via paper flyers left in doctors’ surgeries in the city of Poznań. Subjects who, in their dietary histories, declared dyspeptic symptoms following the consumption of milk and dairy products (such as frequent flatulence, cramps, chronic or recurrent diarrhea, symptoms of gastro-esophageal reflux), as well as asymptomatic volunteers, were invited to participate in the study. 

The study was conducted in accordance with the procedure (Figure 2). 

The type I error probability of α = 0.05 was used to calculate the minimum sample size. The sample size was calculated using Statistica StatSoft 13.3 data analysis software based on a hypothesis test for a difference between the two population means of total serum vitamin D levels (23.3 ng/mL vs. 18.04 ng/mL) with standard deviation (Sigma = 7.09). The calculated minimum sample size was 21 people, with a test power of 0.7167. 

The participants were made aware of the purpose of the study, the manner in which it was going to be conducted, the fact that participation was voluntary and of their option to opt out at any stage without providing a reason. This project and study were in compliance with the Declaration of Helsinki guidelines. This study was carried out pursuant to Approval No. 1109/18 and 1068/19 granted by the Poznań University of Medical Sciences. Written consent, including for blood sampling and genetic testing, was obtained from each participant. The following exclusion criteria were applied: less than 18 years old, systemic diseases (diabetes, kidney and liver diseases, chronic inflammatory conditions), coeliac disease, Crohn’s disease, inflammatory bowel disease, malignancies, vitamin D or calcium metabolism disorders, obesity, pregnancy, antibiotic therapy during the study period and use of corticosteroids. Individuals using vitamin D or calcium supplements were also excluded. Sixty-three individuals between 21 and 31 years of age were included in the study. Eight patients did not meet the inclusion criteria. The study was conducted between November 2019 and March 2020 during the autumn and winter period to minimize the effects of UV-B-induced vitamin D biosynthesis. 

An amount of 10 mL of blood was collected from each participant 12 h after their last meal. The blood samples were taken from an elbow vein venipuncture into an anticoagulant (EDTA) tube at the Central Gynaecological Obstetric Laboratory at the Medical University of Poznań Clinical Hospital. Qualified professional laboratory staff collected the blood samples. All safety precautions were strictly observed whilst collecting blood. The blood obtained for DNA extraction and the serum for biochemical analyses were stored at −80 °C. 

Subjects’ dietary history and genotyping were used to diagnose hypolactasia. 

### 4.2. Genotypes

The incidence of the following gene polymorphism genotypes and allele was determined: LCT-13910 C>T, VDR BsmI (1024 + 283 G > A) and VDR FokI (c.2T > C), using the polymerase chain reaction–restriction fragment length polymorphism method (PCR-RFLP). The 200 µL blood samples were collected for the needs of the study. Genomic DNA was extracted from peripheral blood leukocytes using a blood mini kit from A&A Biotechnology (Poland). The manufacturer’s guidelines were followed in the process. A 7415 nanospectrophotometer (Jenway^®^, Chicago, IL, USA) was used to determine the obtained DNA concentrations. Values of the A260/A280 coefficients within the range of 1.8–2.0. were calculated on the basis of absorbance measurements at wavelengths of 260 and 280 nm. 

Single nucleotide polymorphisms of the LCT and VDR gene were selected using Variation Viewer: https://www.ncbi.nlm.nih.gov/variation/ accessed on 9 June 2023.

DNA was amplified in thermal cycles using the PCR master mix plus reagent (A&A Biotechnology, Gdańsk, Poland). The mix contained optimal concentrations of Taq DNA polymerase, PCR buffer, MgCl_2_, nucleotides and stabilizers to capture polymerization reaction inhibitors, dye and loading buffers, as well as appropriate primers (Laboratory of Sequencing and Oligonucleotide Synthesis (PAS, Warsaw, Poland). Starters were selected on the basis of [80]. Their sequences were additionally checked using the Primer3Plus software [81] (https://www.bioinformatics.nl/cgi-bin/primer3plus/primer3plus.cgi, accessed on 9 June 2023). 

The following starters were used:

For LCT:

- Forward—5′-GCTGGCAATACAGATAAGATAATGGA-3′
- Reverse—5′-CTGCTTTGGTTGAAGCGAAGAT-3′

For VDR BsmI:

- F—5′-GGCAACCAGACTACAAGTACC-3′
- R—5′-TCTTCTCACCTCTAACCAGCG-3′

For VDR FokI:

- F—5′-AGCTGGCCCTGGCACTGACTCTGCTCT-3′
- R—5′-ATGGAAACACCTTGCTTCTTCTCCCTC–3′

PCR reaction conditions to determine the lactose intolerance polymorphism were as follows: initial denaturation: 180 s at 94 °C followed by 35 cycles; denaturation: 45 s at 94 °C; primer binding: 45 s at 58 °C; elongation: 120 s at 72 °C; final synthesis: 300 s at 72 °C.

PCR conditions for VDR polymorphisms were as follows: initial denaturation: 240 s at 94 °C; denaturation: 40 s at 94 °C; primer binding: 40 s at 60 °C for BsmI and at 55 °C for FokI; elongation: 100 s at 72 °C; 31 cycles for BsmI and 35 cycles for FokI; final synthesis: 180 s at 72 °C. Independent PCR reactions were carried out for inconclusive genotypes.

PCR products were digested using the *Hinf I*, *Mva1269I* and *BseGI* restriction enzymes (EURx, Gdańsk, Poland). The procedure was carried out according to the manufacturer’s instructions. It generated fragments of different lengths depending on the presence of a polymorphic restriction site at one or both ends. The resulting digestion products were identified by 3% agarose gel electrophoresis. The results were visualized under UV light and photographed. Genotypes were identified by the presence or absence of an appropriate restriction site. The obtained electrophoresis results were read independently by two people. 

The following genotypes were found for the LCT gene polymorphism: CC (201 bp, no restriction site), CT (201 + 177 + 24 bp) and TT (177 + 24 bp). For the BsmI polymorphism: AA (837 bp, no restriction site), GA (837 + 648 +189 bp) and GG (648 + 189 bp); for FokI: genotype CC (265 bp, no restriction site), CT (265 + 196 + 69 bp) and TT (196 + 69 bp).

### 4.3. Anthropometric Parameters

A body composition analysis using a Tanita MC-780 body composition analyzer (Poland) was carried out for each participant using the bioelectrical impedance method. Body weight, BMI, body fat (%) and water (%), as well as bone mass, were measured. The tests were performed methodologically at the same time of day and at a minimum of 3 h after a meal and physical activity. Measurements were taken using a step-on analyzer. Body height was measured using a measuring rod (Seca, Hamburg, Germany), with an accuracy of up to 0.5 cm.

### 4.4. Assessment of Nutrition

Nutrient intake was assessed on the basis of a dietary history interview conducted in accordance with the guidelines of the Food and Nutrition Institute in Warsaw. As part of the assessment, the respondents, supervised by a dietician, completed a 7-day diet questionnaire. Detailed instructions were given to participants during their first visit. Digital databases developed on the basis of “Food composition table” were used to analyze the results of the qualitative and quantitative questionnaire on the composition of total daily food intake.

The energy values of diets, intake and energy percentage from protein and fat and carbohydrates in daily food portions, as well as estimated intakes of vitamin D, calcium and phosphorus, were determined using the Diet 6.0 certified nutrition software package (Institute of Food and Nutrition, Poland). 

### 4.5. Calcium Intake from Dairy Products

The assessment of the frequency (times/person/day) and amount (g/person/day) of dairy products habitually consumed in the past 6 months and the amount of calcium consumed with dairy products (mg/person/day) and as part of a daily food portion (mg/person/day) was performed using the validated ADOS-Ca questionnaire (a diagnostic test to assess calcium intake) [82].

The core part of the ADOS-Ca questionnaire comprised (closed) questions on the habitual intake frequency and amount for 11 dairy products. These included milk, buttermilk/kefir, hard cheese, fresh cheese, processed cheese, natural yoghurt, fruit yoghurt, cream, ice-cream (during and out of season), homogenized cheese and “Fromage” -type cheese. Multiple choice answers to questions on intake amounts were chosen individually for each product and expressed in common household measures. The ADOS-Ca questionnaire also included questions on current and past nutrition habits, lifestyle and other osteoporosis risk factors. The list of dairy products was developed based on an analysis of the composition of food consumed by Poles. Three classes of calcium intake were established, with 66.7% and 90% of the recommended dietary intake (RDI) amount adopted as safe thresholds: Ca < 66.7% of the recommended amount, 66.7% ≤ Ca < 90% of the recommended amount and Ca ≥ 90% of the recommended amount.

### 4.6. Biochemical Research

#### 4.6.1. Determination of Vitamin D Metabolites

A validated HPLC method (1220 Infinity II, Agilent Technologies, USA) was used to determine vitamin D metabolites (25(OH)D_2_ and 25(OH)D_3_). Separation was carried out in reversed-phase mode with UV detection. Absorbance was measured at λ = 265 nm. Analytes were separated in a LiChroCART^®^250-4 Superspher^®^60R-P-select B analytical column (MERCK, Darmstadt, Germany), 250 mm × 4 mm, load: 3.2 cm^3^. Methanol and water (80:20, *v*/*v*) were used in the mobile phase at a flow rate of 1 cm^3^/min. The 25(OH)D_2_ and 25(OH)D_3_ standards (Santa Cruz Biotechnology, Dallas, TX, USA) were used to generate a standard curve. 

For the determination of vitamin D metabolites, 50 µL of blood serum and 50 µL of internal standard (retinol at 10 µg/mL) were added to 500 µL of 50 g/L human albumin solution. Salting out was performed by adding 350 µL of methanol and propanol mixture (8:2, *v*/*v*), mixing for 30 s and then adding 2000 µL of n-hexane. The samples were mixed and centrifuged for 10 min at 3000× *g*. Then, the top hexane layer was collected. An amount of 2000 µL of n-hexane was added again to the remaining solution. Then, it was centrifuged and the top layer of the solution was collected and combined with the previous one. The organic layer was evaporated at 40 °C under a stream of nitrogen. The resulting residue was dissolved in 120 µL of phase (methanol/water (80:20, *v*/*v*), then it was mixed and centrifuged. An amount of 100 µL was collected and injected into the HPLC system. A linear method over a concentration range of 1–100 ng/mL was used. Method accuracy, expressed by relative standard deviation, was 18.5%. An approximate recovery rate of 72% was obtained for 25(OH)D_2_ and 25(OH)D_3_ from human serum [83]. An example chromatogram of the separation of vitamin D metabolites is presented in the Supplementary Materials (Figure S1). 

#### 4.6.2. Determination of Calcium

Validated atomic absorption spectrometry (iCE 3000 Series, AAS, Thermo Scientific, Cambridge, UK) was used to determine serum calcium concentration. 

Sample dissolution was carried out using a microwave accelerated reaction system (MARS 6, CEM Corporation, Matthews, NC, USA). For this purpose, 1 mL of blood serum was collected in a microwave vessel and 7 mL of 69% ultra-pure nitric acid (ROMIL, Cambridge, UK) was added. The vessels were gently mixed and left open for 15 min to facilitate initial sample digestion. The microwave heating program consisted of two stages: increase from ambient temperature to 180 °C in 20 min and then this temperature was maintained for a further 20 min. After cooling, the resulting solutions were transferred to 50 mL volumetric flasks and diluted to 20 mL with ultra-pure water. All analyses were repeated three times. The correct serum calcium concentration range was between 2.25 and 2.75 mmol/L [84].

### 4.7. Statistical Analysis

Statistica 13.3 software (StatSoft, TIBCO Software Inc., Palo Alto, CA, USA) was used to perform statistical analyses. Descriptive statistics were used to describe the basic nutritional parameters, as well as serum vitamin D and calcium concentrations of the subjects. The Shapiro–Wilk test was used to test the data distribution for normality. The statistical significance of the differences between the study groups of individuals for data with a normal distribution was analyzed using the Student’s *t*-test; the data are presented as means with standard deviations. In the absence of a normal distribution, the Mann–Whitney U test was used and data were presented as medians with interquartile ranges (IQR). Minimum and maximum values were calculated. 

When comparing the significance of differences between multiple groups, a Kruskal–Wallis ANOVA was used (in the absence of a normal distribution) and a Tukey’s RIR post hoc analysis test was used for normal distributions. 

For all tests used, *p* < 0.05 was considered statistically significant. A χ^2^ test was used to test the Hardy–Weinberg equilibrium, as well as allele and genotype frequencies. Pearson’s and Spearman’s correlation coefficient tests were used to determine the correlation between the parameters studied. A multivariate logistic regression model was used to determine the relationship between impaired lactose tolerance and serum vitamin D, as well as calcium status and intake. The odds ratio (OR) and a 95% confidence interval (CI) were calculated.

## 5. Conclusions

To sum up, the -13910 CC genotype associated with hypolactasia significantly affects the consumption of milk and dairy products. Eliminating dairy products from the diet is associated with a reduced intake and vitamin D and calcium deficiency in the Polish population. The mutated A allele of VDR gene BsmI polymorphism present in people with hypolactasia may contribute to an increased risk of vitamin D deficiency. Exclusion of milk sugar from the diet, combined with impaired vitamin D metabolism, may also lead to inhibited calcium absorption by the human body. The body’s calcium and vitamin D status should therefore be monitored. Further studies on a larger number of participants should be carried out to clarify the role of molecular markers of vitamin D and calcium supply status in individuals suffering from hypolactasia.

## Figures and Tables

**Table 1:** Distribution of the LCT (-13910 C>T) genotype and allelic frequencies in the surveyed population.

SNP	Allele/Genotype	Allele Frequency/ Genotypes
	N(%)	
rs4988235	C/T	74/52 (58.7/41.3)
TT	10 (15.9)	
CT	32 (50.8)	
CC	21 (33.3)	

Data presented as absolute value (%), *p* = 0.635, χ^2^—0.076, df2, a chi-square test (χ2) was used to test the Hardy–Weinberg equilibrium. Values < 3.84 were consistent with the H-W equilibrium law.

**Table 2:** The level of selected anthropometric parameters according to the LCT-13910 C>T genotype (CC vs. CT + TT).

Parameters	Lactase Non-Persistence LNP (N = 21)	Lactase Persistence LP (N = 42)	*p*-Value
Male (N %)	10 (16)	13 (21)	-
Female (N %)	11 (17)	29 (46)	-
# Age (years)	23.0 ± 1.50	24.0 ± 1.00	NS
# Height (cm)	174 ± 6.50	172 ± 6.50	NS
Weight (kg)	68.5 ± 12.4	67.7 ± 15.9	NS
BMI (kg/m^2^)	22.8 ± 2.68	22.8 ± 4.63	NS
# Body fat content (%)	21.4 ± 3.37	20.8 ± 6.70	NS
# Bone mass (kg)	2.40 ± 0.25	2.60 ± 0.70	NS
# Body water content (%)	54.5 ± 3.60	55.2 ± 5.20	NS

Mean values ± SD, Student’s *t*-test, # Me ± IQR (interquartile ratio), Mann–Whitney U-test, *p* < 0.05, NS—no statistical differences.

**Table 3:** Intake levels of selected dietary components according to the LCT-13910 C>T genotype (CC vs. CT + TT).

Parameters	LNP (N = 21)	LP (N = 42)	*p*-Value
Male (N %)	10 (16)	13 (21)	-
Female (N %)	11 (17)	29 (46)	-
# Total energy (kcal/d)	2033 ± 542	2094 ± 499	NS
Protein (g/d)	85.8 ± 27.4	84.1 ± 21.9	NS
Protein (% kcal)	17.0 ± 2.58	16.5 ± 1.69	NS
# Fat (g/d)	68.8 ± 16.8	80.3 ± 29.5	NS
Fat (% kcal)	32.7 ± 4.42	34.3 ± 5.32	NS
Carbohydrate (g/d)	253 ± 60.3	262 ± 66.8	NS
Carbohydrate (% kcal)	48.9 ± 4.18	50.4 ± 5.36	NS
# Vitamin D intake (µg/d)	2.32 ±0.36 ^a^	4.57 ± 0.50 ^b^	0.045
# Calcium intake (mg/d)	785 ± 97.5 ^a^	881 ± 128 ^b^	0.048
Phosphorus intake (mg/d)	1357 ± 421	1566 ± 502	NS

Mean values ± SD, Student’s *t*-test, # Me ± IQR, Mann–Whitney U-test, ^a,b^ significant with *p* < 0.05, NS—no statistical differences.

**Table 4:** Estimated average intake of dairy products according to LCT polymorphism.

Parameters	LNP (N = 21)	LP (N = 42)	*p*-Value
Milk (g/d)	134.7 ± 66.7 ^a^	342.5 ± 176 ^b^	0.012
Total dairy products (g/d)	78.50 ± 36.2 ^a^	216.3 ± 102 ^b^	0.008

Me ± IQR, Mann–Whitney U-test, statistically significant differences (*p* < 0.05) are marked with an ^a,b^.

**Table 5:** Vitamin 25(OH)D_2_, 25(OH)D_3_ and 25(OH) (total), as well as calcium serum, levels in the study subjects according to the LCT-13910C>T genotype.

Parameters	**CC** **(N = 21)**	**CT** **(N = 32)**	**TT** **(N = 10)**	***p*-Value**
	Median ± IQR (Min–Max)			
25(OH)D_2_ (ng/mL)	1.42 ± 0.61	1.99 ± 0.36	1.47 ± 0.22	NS
	(1.25–2.40)	(1.20–2.82)	(1.31–2.75)	
25(OH)D_3_ (ng/mL)	17.6 ± 2.19 ^a^	19.5 ± 2.79 ^ab^	21.5 ± 3.99 ^b^	0.025
	(8.58–23.9)	(9.26–32.6)	(16.8–43.7)	
Total (D_2_+D_3_) (ng/mL)	18.1 ± 2.91 ^a^	20.8 ± 2.97 ^ab^	22.6 ± 4.46 ^b^	0.022
	(9.58–24.8)	(9.28–33.0)	(16.9–44.1)	
	Mean values ± SD (Min–Max)			
* Calcium (mg/dL)	2.27 ± 0.29 ^a^	2.56 ± 0.35 ^ab^	2.62 ± 0.26 ^b^	0.049
	(1.76–2.75)	(1.57–3.17)	(2.28–3.02)	

No normal distribution: Kruskal–Wallis ANOVA, * normal distribution: post hoc Tukey’s RIR test. Values marked ^a,b^ are statistically significantly different, *p* < 0.05.

**Table 6:** Relationship between lactase non-persistence (CC genotype) and serum total vitamin D and calcium concentrations and their intake levels.

Variables	Raw Structure Model	
	*p*-Value	Adjusted OR (95% Cl)
Total vitamin D		
(D2 + D3) in serum	0.035	1.21 (0.92; 1.40)
Calcium in serum	0.022	1.15 (0.84; 1.23)
Vitamin D intake	0.040	1.11 (1.05; 1.37)
Calcium intake	0.032	1.20 (0.98; 1.28)

OR—odds ratio; Cl—confidence interval.

**Table 7:** Allele and genotype frequencies of VDR BsmI (1024 + 283 G > A) and VDR FokI (c.2T > C) in the study group.

SNP	Allele/Genotype	Allele Frequency/ Genotypes
	N (%)	
BsmI (rs1544410)	G/A	64/62 (50.8/49.2)
GG	15 (23.8)	
GA	34 (54.0)	
AA	14 (22.2)	
FokI (rs2228570)	T/C	49/77 (38.9/61.1)
TT	12 (19.0)	
TC	25 (39.7)	
CC	26 (41.3)	

Data presented as absolute value (%), a chi-square test (χ^2^) was used to test the Hardy–Weinberg equilibrium. Values < 3.84 were consistent with the H-W equilibrium law.

**Table 8:** Distribution of VDR genotypes and allelic frequencies in dependence on lactase persistence.

VDR Genotypes	LNP (N = 21)	LP (N = 42)	*p*-Value
	N (%)		
BsmI			
GG	6 (28.6)	9 (21.4)	NS
GA	10 (47.6)	24 (57.2)	NS
AA	5 (23.8)	9 (21.4)	NS
FokI			
TT	8 (38.1)	8 (19.0)	NS
TC	9 (42.9)	16 (38.1)	NS
CC	4 (19.0)	18 (42.9)	0.042

The significance of difference was analyzed at *p* < 0.05; NS—no statistical differences.

**Table 9:** χ^2^ value of the tested VDR gene polymorphisms in the LNP group and the LP control group.

Polymorphism	LNP	LP
BsmI	0.05	0.85
FokI	0.23	1.41

A chi-square test (χ^2^) was used to test the Hardy–Weinberg equilibrium. Values < 3.84 were consistent with the H-W equilibrium law.

**Table 10:** Vitamin D (total) and serum calcium levels according to VDR genotype (BsmI and FokI) and the LCT-13910 C>T genotype.

SNP	LNP		LP	
	Total Vitamin D (ng/mL) (Min–Max)	Calcium (mg/dL) (Min–Max)	Total Vitamin D (ng/mL) (Min–Max)	Calcium (mg/dL) (Min–Max)
BsmI				
GG	21.6 ± 0.67 ^a^	2.46 ± 0.26 ^a^	22.6 ± 3.53 ^a^	2.50 ± 0.26
	(19.3–24.9)	(2.05–2.75)	(9.56–43.7)	(2.21–3.07)
GA	18.1 ± 1.99 ^ab^	2.38 ± 0.37 ^ab^	19.3 ± 2.76 ^ab^	2.71 ± 0.28
	(10.6–23.4)	(1.87–2.75)	(9.26–32.6)	(2.0–3.17)
AA	10.4 ± 1.25 ^b^	2.28 ± 0.12 ^b^	17.0 ± 2.79 ^b^	2.33 ± 0.32
	(9.58–16.8)	(2.11–2.43)	(12.2–23.7)	(1.60–2.70)
*p*-value	0.017	0.049	0.048	NS
FokI				
CC	13.2 ± 2.16 ^a^	2.04 ± 0.06 ^a^	20.5 ± 5.19	2.17 ± 0.26 ^a^
	(10.4–22.4)	(1.96–2.11)	(9.56–28.7)	(1.57–2.38)
TC	19.7 ± 1.30 ^b^	2.52 ± 0.21 ^b^	21.8 ± 2.36	2.55 ± 0.24 ^b^
	(12.3–24.9)	(2.13–2.75)	(10.0–32.6)	(2.21–3.17)
TT	17.2 ± 2.29 ^b^	2.37 ± 0.31 ^b^	20.0 ± 2.43	2.70 ± 0.21 ^c^
	(9.58–20.8)	(1.87–2.75)	(9.26–43.7)	(2.37–3.07)
*p*-value	0.05	0.05	NS	0.036

Me ± IQR and mean values ± SD. No normal distribution: Kruskal–Wallis ANOVA; normal distribution: post hoc Tukey’s RIR test. ^a,b,c^ significant with *p* < 0.05, NS—no statistical differences.

**Table 11:** Anthropometric parameters, intake levels of selected nutrients and serum calcium levels and VDR polymorphisms (BsmI and FokI) according to LCT-13910 C>T genotype and serum vitamin D levels (deficiency, subclinical and optimal levels).

Parameters	LNP			LP			
	Total Vitamin D in Serum						
	<20 ng/mL	>20 <30 ng/mL	*p*-Value	<20 ng/mL	>20 <30 ng/mL	>30 ng/mL	*p*-Value *
Weight (kg)	68.4 ± 10.8	68.9 ± 16.9	NS	63.5 ± 13.7	71.2 ± 17.7	69.0 ± 12.6	NS
BMI (kg/m^2^)	23.0 ± 2.46	22.4 ± 3.38	NS	21.5 ± 3.27	23.8 ± 5.61	22.9 ± 2.53	NS
# Bone mass (kg)	2.30 ± 0.40	2.50 ± 0.25	NS	2.35 ± 0.15	2.50 ± 0.45	2.80 ± 0.55	NS
# Total energy (kcal/d)	2022 ± 277	1638 ± 380	NS	1884 ± 453	2239 ± 466	1933 ± 380	NS
Protein (g/d)	85.4 ± 15.3	64.6 ± 16.1	NS	85.1 ± 22.9	84.9 ± 22.3	72.4 ± 13.5	NS
Fat (g/d)	70.0 ± 17.3	64.1 ± 18.0	NS	81.7 ± 14.9	79.2 ± 14.1	75.9 ± 14.1	NS
Carbohydrate ((g/d)	258 ± 48.4	241 ± 58.0	NS	260 ± 67.0	266 ± 70.3	245 ± 57.1	NS
# Vitamin D intake (µg/d)	2.23 ± 0.72 ^a^	3.07 ± 0.51 ^b^	0.050	3.92 ± 1.22 ^a^	4.37 ± 0.84 ^ab^	5.13 ± 0.89 ^b^	0.048
# Calcium intake (mg/d)	812 ± 133	830 ± 65.9	NS	899 ± 144	915 ± 175	1018 ± 173	NS
Phosphorus intake (mg/d)	1398 ± 369	1256 ± 457	NS	1528 ± 449	1619 ± 570	1419 ± 361	NS
Calcium (mg/dL)	2.28 ± 0.29	2.32 ± 0.14	NS	2.39 ± 0.38	2.57 ± 0.30	2.58 ± 0.17	NS
BsmI (N%)							
GG	1 (4.76)	5 (23.8)	NS	2 (4.76)	6 (14.3)	2 (4.76)	NS
GA	9 (42.9)	1 (4.76)	NS	12 (28.6)	11 (26.2)	1 (2.38)	NS
AA	5 (23.8)	0	-	4 (9.52)	2 (4.76)	0	NS
FokI (N%)							
CC	3 (14.3)	1 (4.76)	NS	4 (9.52)	2 (4.76)	2 (4.76)	NS
TC	5 (23.8)	4 (19.0)	NS	5 (11.9)	6 (14.3)	5 (11.9)	NS
TT	7 (33.3)	1 (4.76)	NS	1 (2.38)	15 (35.7)	2 (4.76)	NS

# Me ± IQR and mean values ± SD. No normal distribution: Mann–Whitney U test; normal distribution: Student’s *t*-test. * No normal distribution: Kruskal–Wallis ANOVA; normal distribution: post hoc Tukey’s RIR test. ^a,b^ significant with *p* < 0.05. NS—no statistical differences.

## Data Availability

The data presented in this study are available on request from the corresponding author. The data are not publicly available due to privacy protection.

## Supplementary Materials

The supporting information can be downloaded at: https://www.mdpi.com/article/10.3390/ijms241210191/s1.
