# Supplementary figures and images for: A Comprehensive Look at the -13910 C>T LCT Gene Polymorphism as a Molecular Marker for Vitamin D and Calcium Levels in Young Adults in Central and Eastern Europe: A Preliminary Study
Source: Int J Mol Sci. 2023 Jun 15;24(12):10191. doi: 10.3390/ijms241210191 (PMC10298876; doi:10.3390/ijms241210191)

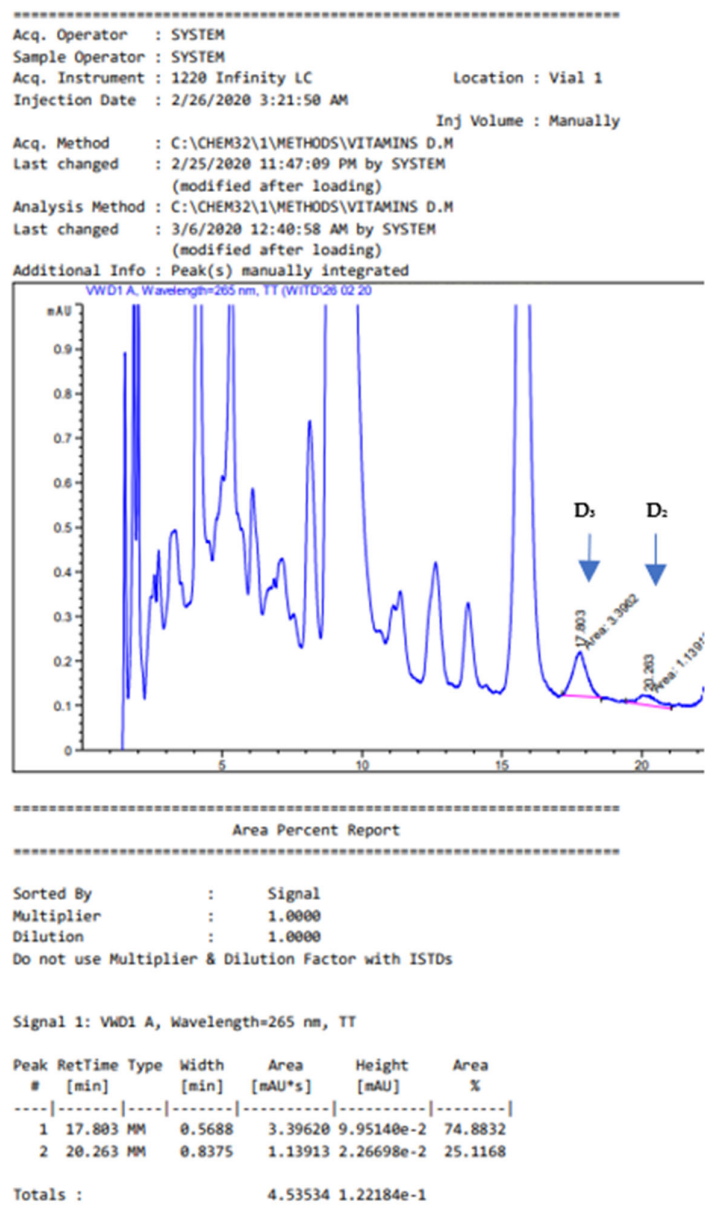

**Figure S1.** An example chromatogram of the separation of vitamin D metabolites.

Supplement: Supplementary file 1 [file ijms-24-10191-s001.zip › ijms-2438454-supplementary.pdf]
